# SREBP2 regulates the endothelial response to cytokines via direct transcriptional activation of *KLF6*

**DOI:** 10.1016/j.jlr.2023.100411

**Published:** 2023-07-10

**Authors:** Joseph Wayne M. Fowler, Nabil E. Boutagy, Rong Zhang, Daiki Horikami, Michael B. Whalen, Casey E. Romanoski, William C. Sessa

**Affiliations:** 1 Department of Pharmacology, Vascular Biology and Therapeutics Program, Yale University School of Medicine, New Haven, CT, USA; 2 Department of Cellular and Molecular Medicine, The University of Arizona, Tucson, AZ, USA

**Keywords:** Endothelium, inflammation, nuclear receptors/SREBP, cholesterol, chemokines, interferon

## Abstract

The transcription factor SREBP2 is the main regulator of cholesterol homeostasis and is central to the mechanism of action of lipid-lowering drugs, such as statins, which are responsible for the largest overall reduction in cardiovascular risk and mortality in humans with atherosclerotic disease. Recently, SREBP2 has been implicated in leukocyte innate and adaptive immune responses by upregulation of cholesterol flux or direct transcriptional activation of pro-inflammatory genes. Here, we investigate the role of SREBP2 in endothelial cells (ECs), since ECs are at the interface of circulating lipids with tissues and crucial to the pathogenesis of cardiovascular disease. Loss of *SREBF2* inhibits the production of pro-inflammatory chemokines but amplifies type I interferon response genes in response to inflammatory stimulus. Furthermore, SREBP2 regulates chemokine expression not through enhancement of endogenous cholesterol synthesis or lipoprotein uptake but partially through direct transcriptional activation. Chromatin immunoprecipitation sequencing of endogenous SREBP2 reveals that SREBP2 bound to the promoter regions of two nonclassical sterol responsive genes involved in immune modulation, *BHLHE40* and *KLF6*. SREBP2 upregulation of KLF6 was responsible for the downstream amplification of chemokine expression, highlighting a novel relationship between cholesterol homeostasis and inflammatory phenotypes in ECs.

## Graphical abstract

**Figure:**
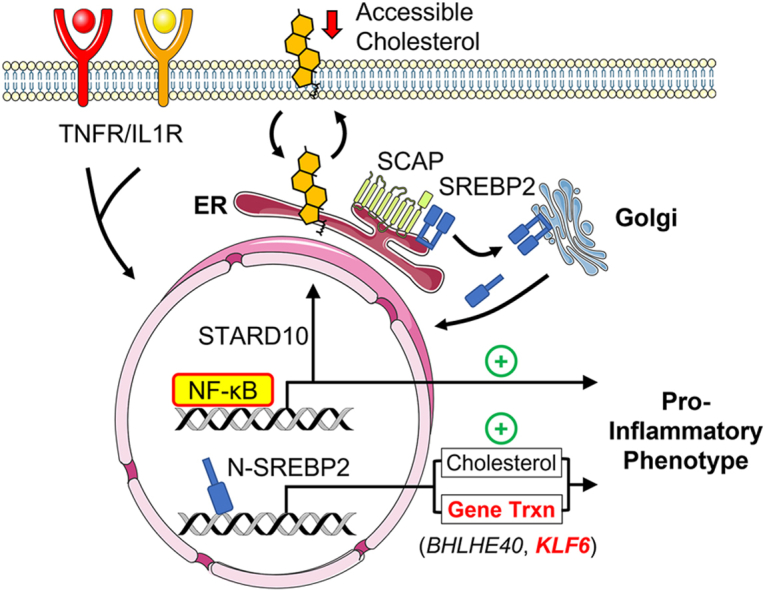

There is a growing appreciation that cellular metabolism and immune phenotype are deeply intertwined. This field of study, coined immunometabolism, is centered around the idea that metabolic disturbances can alter leukocyte phenotype (1). Unlike glucose or fatty acids, cholesterol cannot be utilized for the generation of ATP. However, cholesterol is an essential molecule needed for the formation of cellular membranes, overall cell growth, and biosynthesis of steroids. Additionally, cholesterol shares several of the same regulatory mechanisms as fatty acids, namely by the transcription factor, sterol response element binding protein (SREBP2). SREBP2 directly transcribes genes necessary for de novo cholesterol biosynthesis and exogenous cholesterol uptake (2). It is under the control of a tight negative feedback loop, whereby under conditions of low cellular cholesterol, the adaptor protein, SREBP cleavage-activating protein (SCAP), shuttles SREBP2 from the ER to the Golgi for proteolytic cleavage and release of the DNA-binding N-terminal domain into the nucleus (3). When cellular cholesterol stores are sufficient, SREBP2 is locked in the ER, preventing transcriptional activity. Several reports have indicated that cholesterol flux and SREBP2 play key roles in leukocyte immunobiology (4, 5, 6, 7, 8, 9).

Much of the literature exploring cholesterol homeostasis and cellular phenotype has centered on leukocytes and few have looked at other important cell types, such as ECs. However, there have been reports that increased SREBP2 and cholesterol in ECs are pro-inflammatory, especially in the context of atherosclerosis. Endothelial activation and dysfunction are thought to be the key initiating drivers of atherosclerosis, and the disease, in general, is characterized by two pathologies: hypercholesterolemia and inflammation (10). ECs exposed to pro-atherogenic oscillatory shear stress upregulated SREBP2, which was then shown to transcriptionally activate components of the inflammasome, NACHT, LRR, and PYD domains-containing protein 3 (*NLRP3*) and NADPH oxidase 2 (*NOX2*) (11). The inflammasome and EC inflammation have also been reported to be elevated by the accumulation of LDL in ECs and subsequent cholesterol crystal formation (12, 13). Inflammatory-mediated upregulation of SREBP2 could hypothetically overload ECs with cholesterol, which could be compounded by the exceptionally hyperlipidemic and pro-inflammatory microenvironment of ECs surrounding an atheroma. Another study inhibited the cholesterol efflux pathway via endothelial-specific knockout of phospholipid-transporting ATPase 1 (*Abca1*) and ATP-binding cassette sub-family G member 1 (*Abcg1*), which resulted in an accumulation of EC cholesterol (14). Knockout mice showed evidence of increased vascular inflammation, decreased nitric oxide activity, and exacerbated atherosclerosis. Altogether, cholesterol homeostasis has been implicated to play a role in EC function, but much of the detailed mechanisms regulating this relationship remain to be explored.

Our previous report established a clear relationship between cholesterol homeostasis and EC inflammatory stress (15). We found that inflammatory cytokines mediate the depletion of accessible cholesterol via NF-κB transcriptional regulation of novel genes, including START domain-containing protein 10 (*STARD10*). This decrease in accessible cholesterol enhanced the activation of SREBP2 cleavage and transcriptional activity within the later stages of the acute inflammatory response. In this study, we examine the role of the SREBP2 pathway in the overall endothelial inflammatory phenotype. We characterize the EC transcriptomic response to the loss of SREBP2 combined with cytokine treatment and show that SREBP2 regulates a particular subset of genes in the late phase of acute inflammatory stress. Furthermore, we find that non-classical SREBP2 transcriptional activity likely accounts for this phenotype instead of an obvious effect on cholesterol homeostasis. Endogenous SREBP2 ChIP-seq reveals significant binding to the promoter region of two pro-inflammatory mediators, class E basic helix-loop-helix protein 40 (*BHLHE40*) and Krueppel-like factor 6 (*KLF6*). We validate that SREBP2 directly activates both genes and that the knockdown of *KLF6* phenocopies the transcriptomic response to *SREBF2* knockdown in the context of EC inflammatory stress.

## Materials and methods

### Mammalian cell culture

HUVECs were obtained from the Yale School of Medicine, Vascular Biology, and Therapeutics Core facility. Cells were cultured in EGM-2 media (Lonza) with 10% fetal bovine serum (FBS), penicillin/streptomycin, and glutamine (2.8 mM) in a 37°C incubator with 5% CO2 supply.

### RNA sequencing

RNA was isolated using the RNeasy Plus Kit (Qiagen), and the purity of total RNA per sample was verified using the Agilent Bioanalyzer (Agilent Technologies, Santa Clara, CA). RNA sequencing was performed through the Yale Center for Genome Analysis using an Illumina HiSeq 2000 platform (paired-end 150 bp read length). Briefly, rRNA was depleted from RNA using Ribo-Zero rRNA Removal Kit (Illumina). RNA libraries were generated from control cells using TrueSeq Small RNA Library preparation (Illumina) and sequenced for 45 cycles on Illumina HiSeq 2000 platform (paired-end, 150 bp read length).

### RNA-seq analysis

Normalized counts and gene set enrichment analysis statistics were generated with Partek Flow. Reads were aligned to the hg19 build of the human genome with STAR and quantified to an hg19 RefSeq annotation model through Partek E/M. Gene counts were normalized as counts per million (CPM) and differential analysis was performed with GSA. Ingenuity Pathway Analysis (Ingenuity Systems QIAGEN) software was used to perform Canonical Pathway and Upstream Regulator analyses (Cutoff: *P* < 0.05; −1.5>Fold Change>1.5). Data are deposited in NCBI Gene Expression Omnibus and are available under GEO accession GSE207787 and GSE207919.

### Western blotting analysis

Cells or tissues were lysed on ice with ice-cold lysis buffer containing 50 mM Tris-HCl, pH 7.4, 0.1 mM EDTA, 0.1 mM EGTA, 1% Nonidet P-40, 0.1% sodium deoxycholate, 0.1% SDS, 100 mM NaCl, 10 mM NaF, 1 mM sodium pyrophosphate, 1 mM sodium orthovanadate, 1 mM Pefabloc SC, and 2 mg/ml protease inhibitor mixture (Roche Diagnostics) and samples prepared. Total protein (25 μg) was loaded into SDS-PAGE followed by transfer to nitrocellulose membranes. Immunoblotting was performed at 4°C overnight followed by 1 h incubation with LI-COR compatible fluorescent-labeled secondary antibodies (LI-COR Biosciences). Bands were visualized on the Odyssey CLx platform (LICOR Biosciences). Quantifications were based on densitometry using ImageJ.

### Quantitative RT-qPCR

RNA from cells or tissues was isolated using the RNeasy Plus Kit (Qiagen). 0.5 mg RNA/sample was retrotranscribed with the iScript cDNA Synthesis Kit (BioRad). Real-time quantitative PCR (qPCR) reactions were performed in duplicate using the CFX-96 Real-Time PCR system (Bio-Rad). Quantitative PCR primers were designed using Primer3 software and synthesized by the Yale School of Medicine Oligo Synthesis facility. Fold changes were calculated using the comparative Ct method.

### ALOD4 purification

ALOD4 construct was generously provided by the lab of Dr Arun Radhakrishnan. Recombinant His-tagged ALOD4 and OlyA were purified as previously described (16). Briefly, ALOD4 expression was induced with 1 mM IPTG in OD_0.5_ BL21 (DE3) pLysS *E. coli* for 16 h at 18°C. Cells were lysed and His-ALOD4 and His-OlyA were isolated by nickel purification followed by size exclusion chromatography (HisTrap-HP Ni column, Tricorn 10/300 Superdex 200 gel filtration column; FPLC AKTA, GE Healthcare). Protein-rich fractions were pooled and concentration was measured using a NanoDrop instrument.

### ALOD4 binding and Western blot analysis

At time of collection, HUVEC were washed three times for 5 min in PBS with Ca^2+^ and Mg^2+^ containing 0.2% (wt/vol) BSA. Cells were then incubated with 3 μM ALOD4 in basal EBM2 media containing 0.2% (wt/vol) BSA for 1 h at 4°C. The unbound proteins were removed by washing three times with PBS with Ca^2+^ and Mg^2+^ for 5 min each. Cells were then lysed and prepared for SDS-PAGE and immunoblotting. ALOD4 was probed on nitrocellulose gels using anti-6X His (Abcam) antibody at 15 kDa. A similar method was used for OlyA binding.

### Cytokine/chemokine ELISA

Media was collected from treated cells and stored in −80°C until the time of assay. Media was diluted 1:100 in basal medium, and an assay was performed according to kit provided protocol (R&D Systems).

### Chromatin immunoprecipitation sequencing

Chromatin immunoprecipitation sequencing (ChIP-seq) was performed as previously described (17). Briefly, HUVEC were fixed at room temperature with 2 mM disuccinimidyl glutarate (DSG) for 30 min before an additional fixation with 1% formaldehyde for 15 min and quenched with glycine. Between 5 and 15 million cells were used for each ChIP-seq. Cell lysates were sonicated using a BioRuptor Standard or BioRuptor Pico (Diagenode, Belgium), and then immunoprecipitated using antibodies bound to a 2:1 mixture of Protein A Dynabeads (Invitrogen #10002D) and Protein G Dynabeads (Invitrogen #10004D). A cocktail of antibodies targeting the N-terminal domain of SREBP2 were used for SREBP2 pull-down (Sigma #MABS1988, Novus #NBP1-54446, Abcam #ab30682). Following immunoprecipitation, crosslinking was reversed and libraries were prepared using dsDNA end repair and excluding UDG. For each sample condition, an input library was also created using an aliquot of sonicated cell lysate that had not undergone immunoprecipitation. These samples were sequenced and used to normalize ChIP-seq results. Libraries were sequenced on an Illumina HiSeq 4000 according to the manufacturer’s specifications at the University of California–San Diego and the University of Chicago. Reads from ChIP-seq were mapped to the hg19 build of the human genome with Bowtie2. Data for ChIP-seq analysis is available in GEO under accession code GSE223094.

### Flow cytometry

Cells were cultured in 12-well plates, treated, and suspended in Ca^2+^ and Mg^2+^ containing 2% FBS and washed 3 times. Cells were stained with FITC-ICAM1, PE-VCAM1, and PacificBlue-HLA-A,B,C (Biolegend). Cells were washed three times before fixation in 4%PFA. Mean fluorescence intensity per cell was measured by LSRII (BD Biosciences) flow cytometer the next day and data was analyzed using FlowJo.

### Lentiviral-mediated expression

pSMPP lentiviral transfer plasmid empty backbone (Addgene, #104970) was used for the expression of cDNA into HUVEC. FLAG-SREBP2 was inserted into the lentiviral vector via NEBuilder HiFi DNA Assembly (NEB, #E2621) using previously generated cDNA (Addgene, #26807).

### Statistics

Statistical differences were measured with an unpaired two-sided Student’s *t* test or ANOVA with listed corrections for multiple corrections. A value of *P* < 0.05 was considered statistically significant. “n” within figure legends involving HUVEC denotes the number of donors used for the respective experiment. Data analysis was performed with GraphPad Prism software (GraphPad, San Diego, CA).

### Cell lines

The HUVECs used in this study were primary isolates of ECs collected from donor umbilical cords through Yale School of Medicine’s Vascular Biology and Therapeutic Core as described previously (18). No transformations were performed and they were used at low passage (p4).

## Results

### SREBP2 regulates a specific transcriptomic phenotype in EC

**Fig. 1:**
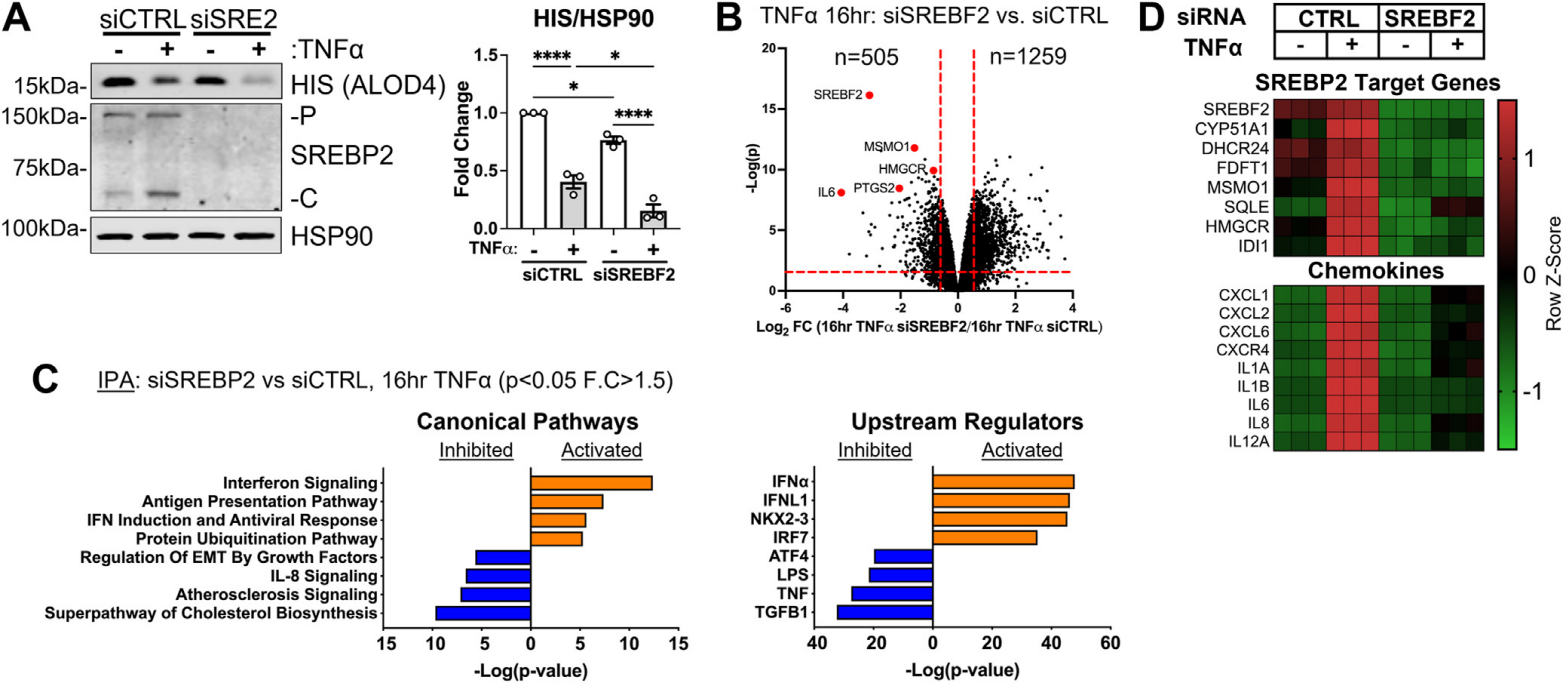
Loss of SREBP2 inhibits the transcription of pro-inflammatory chemokines and upregulates expression of type I interferon signaling in TNFα-treated ECs. A: immunoblot of ALOD4, SREBP2-P (precursor), and SREBP2-C (cleaved) protein levels in HUVEC treated with *SREBF2* siRNA (siSRE2) and with or without TNFα (10 ng/ml). Data are normalized to respective HSP90 levels and then to untreated cells (n = 3). B: volcano plot of RNA-seq analysis of differentially expressed genes of HUVEC treated with TNFα (10 ng/ml) for 16 h and with or without siRNA targeting *CTRL* or *SREBF2*. Red lines indicate cutoffs used for pathway analysis (−1.5<F.C.<1.5; *P* < 0.05). C: ingenuity pathway analysis for pathways and upstream regulators using genes from (B). D: Heatmap of representative genes from (B) showing three independent donors. Data represent the analysis of three independent donor lines. Data are available in GEO under accession code GSE207787. ∗*P* < 0.05; ∗∗*P* < 0.01; ∗∗∗*P* < 0.001; ∗∗∗*P* < 0.0001 by one-way ANOVA with Tukey’s multiple comparison test.

Our previous study indicated that SREBP2 was activated in the late phase of EC acute inflammatory responses due to a decrease in accessible cholesterol (15). Therefore, we evaluated the effect of SREBP2 loss on the EC transcriptome during TNFα treatment. Knockdown of *SREBF2* efficiently decreased total SREBP2 protein levels and decreased basal levels of accessible cholesterol quantified by ALOD4 binding (Fig. 1A). As expected, 16 h TNFα treatment combined with *SREBF2* silencing severely decreased accessible cholesterol because the cells were unable to replenish cholesterol via de novo synthesis or exogenous uptake. RNA sequencing analysis of 16 h TNFα-treated ECs revealed that 505 genes were significantly downregulated and 1,259 genes were significantly upregulated with the loss of SREBP2 (*P* < 0.05, −1.5 > Fold Change (F.C) > 1.5) (Fig. 1B). As anticipated, IPA Canonical Pathway analysis revealed that the “Superpathway of Cholesterol Biosynthesis” was the most significantly downregulated pathway when SREBP2 was silenced (Fig. 1C). Loss of SREBP2 also significantly decreased expression of genes belonging to the pro-inflammatory pathways “Atherosclerosis Signaling” and “IL8 Signaling,” as well as inhibited the predicted upstream regulators “TNF” and “LPS.” These data sets included several genes that belonged to chemokine signaling and chemoattraction, such as interleukin-6 (*IL6*), C-X-C motif chemokine 1 (*CXCL1*), C-X-C motif chemokine 8 (*CXCL8*), and C-X-C motif chemokine receptor type 4 (*CXCR4*) (Fig. 1D). Furthermore, SREBF2 knockdown significantly increased pathways involved in type I interferon signaling and MHC class I presentation, which has been previously reported in macrophages (6).

### Inactivation of SREBP2 by independent methods attenuates the expression and protein secretion of pro-inflammatory chemokines IL6 and CXCL8

**Fig. 2:**
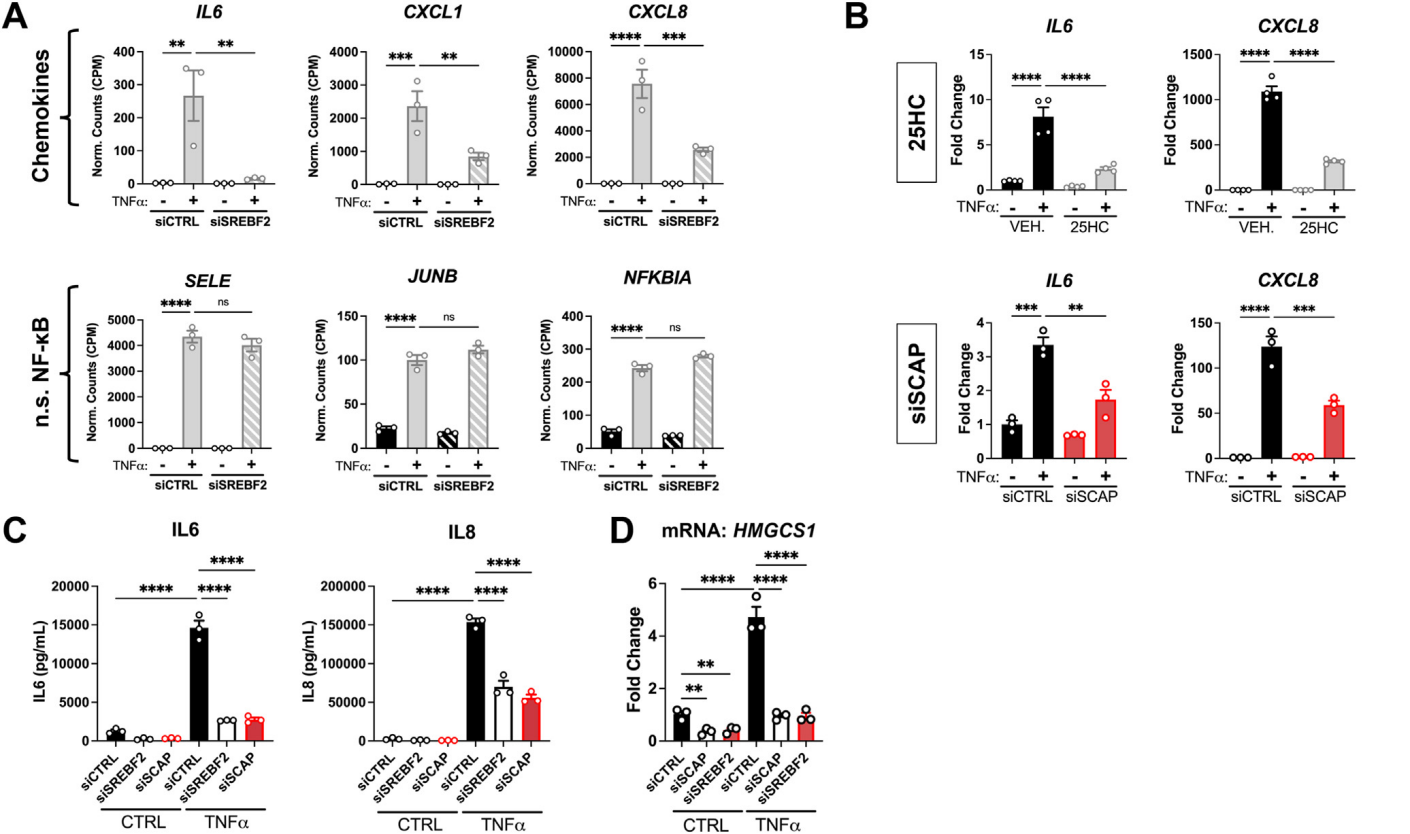
SREBP2 inhibition significantly attenuates IL6 and IL8 mRNA levels and protein expression. A: expression of chemokines that are altered with SREBF2 knockdown as well as several classical NF-κB genes that are unaffected by loss of *SREBF2* from previous RNA-seq experiment. (n = 3). B: qRT-PCR analysis of RNA from HUVEC treated with 25-hydroxycholesterol (25HC) (10 μM) or siSCAP and with or without TNFα (10 ng/ml). HUVEC were treated with siRNA for 48 h prior to 16 h treatment with 25HC in full serum medium. Data are normalized to respective *ACTB* and then to untreated cells (n = 3). C: IL6 and IL8 ELISA from media collected from HUVEC treated with siRNA against *SREBF2* or *SCAP* and with or without TNFα (10 ng/ml). (n = 3). D: qPCR analysis of mRNA from HUVEC treated with similar conditions as in (C). *HMGCS1* is a key downstream target of SREBP2 and serves as a surrogate readout of SREBP2 activity. ∗*P* < 0.05; ∗∗*P* < 0.01; ∗∗∗*P* < 0.001; ∗∗∗*P* < 0.0001 by one-way ANOVA with Tukey’s multiple comparison test.

We validated that the inhibited genes identified from RNA-seq with *SREBF2* knockdown translated to changes in protein and that the results could be recapitulated through other modes of SREBP2 inhibition. As previously mentioned, the gene expression of several chemokines, such as *IL6*, *CXCL1*, and *CXCL8*, was inhibited by the loss of SREBP2 (Fig. 2A). These chemokines are important for the recruitment, activation, and extravasation of leukocytes to the site of injury detected by ECs (19). Notably, *SREBF2* knockdown did not affect several other classical NF-κB genes, such as E-selectin (*SELE*), transcription factor JunB (*JUNB*), and NF-kappa-B inhibitor alpha (*NFKBI*), suggesting that SREBP2 controls a distinct pathway of the EC inflammatory phenotype. To confirm these RNA-seq results, SREBP2 was inactivated by 25-hydroxycholesterol (25HC) and siRNA silencing of *SCAP* (Fig. 2B). Our previous results revealed that 25HC treatment and SCAP knockdown were both sufficient to significantly inhibit SREBP2 activity, even in the presence of TNFα (15). Both treatments significantly reduced *IL6* and *CXCL8* expression in TNFα-treated cells. Second, we confirmed that the decrease in chemokine transcripts reflected protein levels. *SREBF2* knockdown significantly reduced both IL6 and IL8 protein levels in the media of TNFα-treated ECs (Fig. 2C). Loss of SCAP inhibited IL6 and IL8 to similar levels as *SREBF2* silencing and both treatments significantly reduced expression of the sterol-responsive downstream target hydroxymethylglutaryl-CoA synthase 1 (*HMGCS1*) (Fig. 2D). These results indicated that SREBP2 feeds forward to promote a pro-inflammatory phenotype in EC.

**Fig.3:**
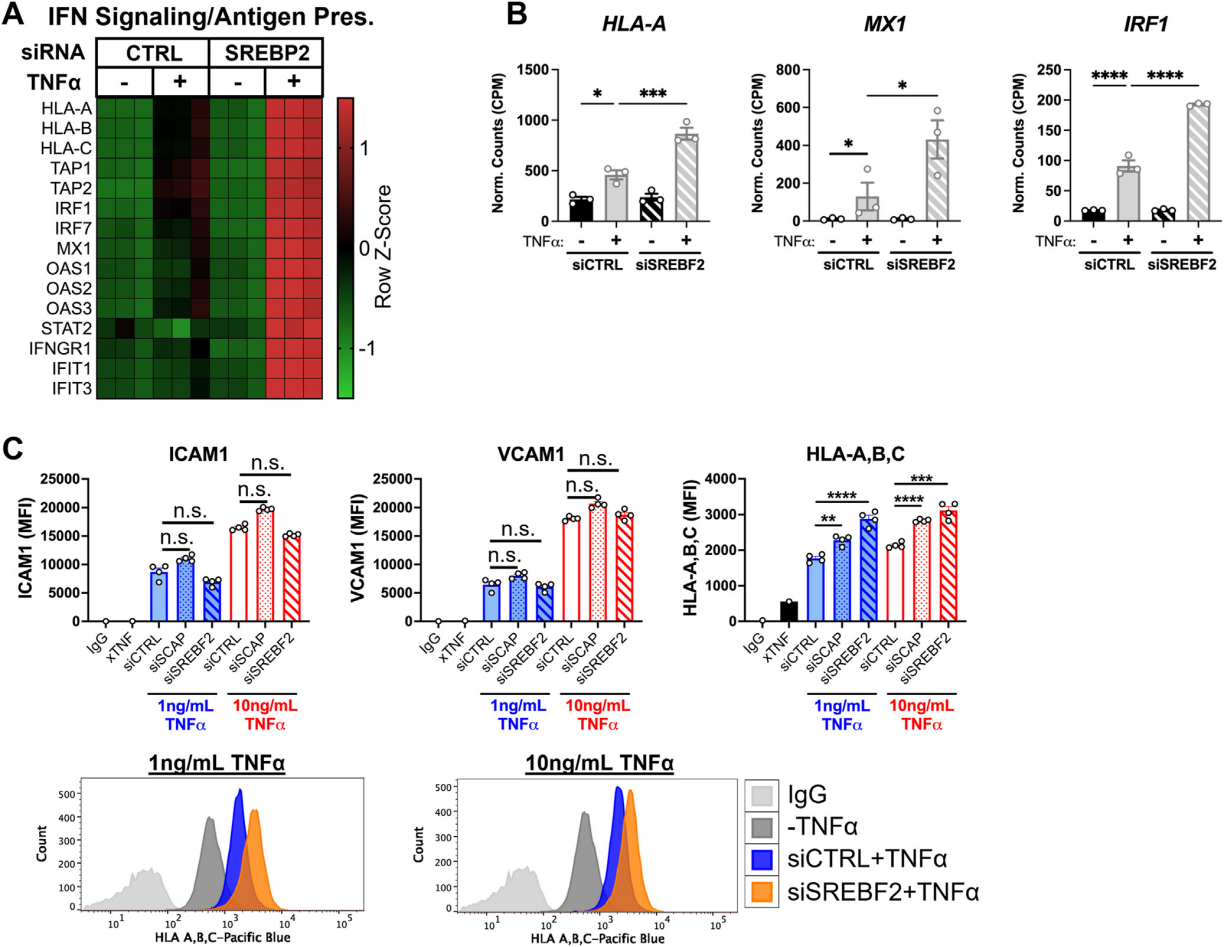
*SCAP* and *SREBF2* knockdown significantly increase type I interferon response genes and protein surface expression. A: heatmap of representative genes from Fig. 1B showing three independent donors. B: *HLA*-*A*, *MX1*, and *IRF1* expression from RNA-seq experiment. C: flow cytometry analysis of surface ICAM1, VCAM1, or HLA-A,B,C levels in HUVEC treated with *siSCAP* or *siSREBF2* at the indicated dose of TNFα. Expression was quantified as the mean fluorescence intensity of the respective fluorophore per cell (10,000 events/replicate, n = 3). ∗*P* < 0.05; ∗∗*P* < 0.01; ∗∗∗*P* < 0.001; ∗∗∗*P* < 0.0001 by either one-way ANOVA with Tukey’s multiple comparison test (A) or two-way ANOVA with Sidak’s multiple comparisons test (B).

Of note, IPA revealed that genes belonging to the type I interferon signaling pathway were significantly elevated in ECs treated with TNFα and *SREBF2* siRNA. These genes play important roles in host defense, such as the presentation of intracellular antigen (*HLA*-*A*, *B*, and *C*), viral RNA/DNA inhibitors (*MX1*), and positive feedback into the type I interferon response (*IRF1*) (Fig. 3, A and B) (20). TNFα has been reported to upregulate these factors in ECs (17). We further validated the RNA-seq results by analyzing surface expression of adhesion molecules and the MHC class I complex. Cells were treated with either 1 ng/ml or 10 ng/ml of TNFα and surface expression of ICAM1, VCAM1, and HLA-A,B,C were simultaneously measured by flow cytometry. Neither knockdown of *SCAP* or *SREBF2* significantly affected the surface expression of NF-κB targets, ICAM1 and VCAM1, which also did not significantly change in our RNA-seq results (Fig. 3C). However, loss of *SCAP* or *SREBF2* significantly increased HLA-A,B,C expression at both doses of TNFα. This indicates that SREBP2 may regulate a specific phenotypic signature in the late-phase EC inflammatory response that includes positive regulation of chemokine production and suppression of the type I interferon pathway.

### Upregulation of SREBP2 activity via LDLR and HMGCR knockdown increases proinflammatory chemokine expression

**Fig. 4:**
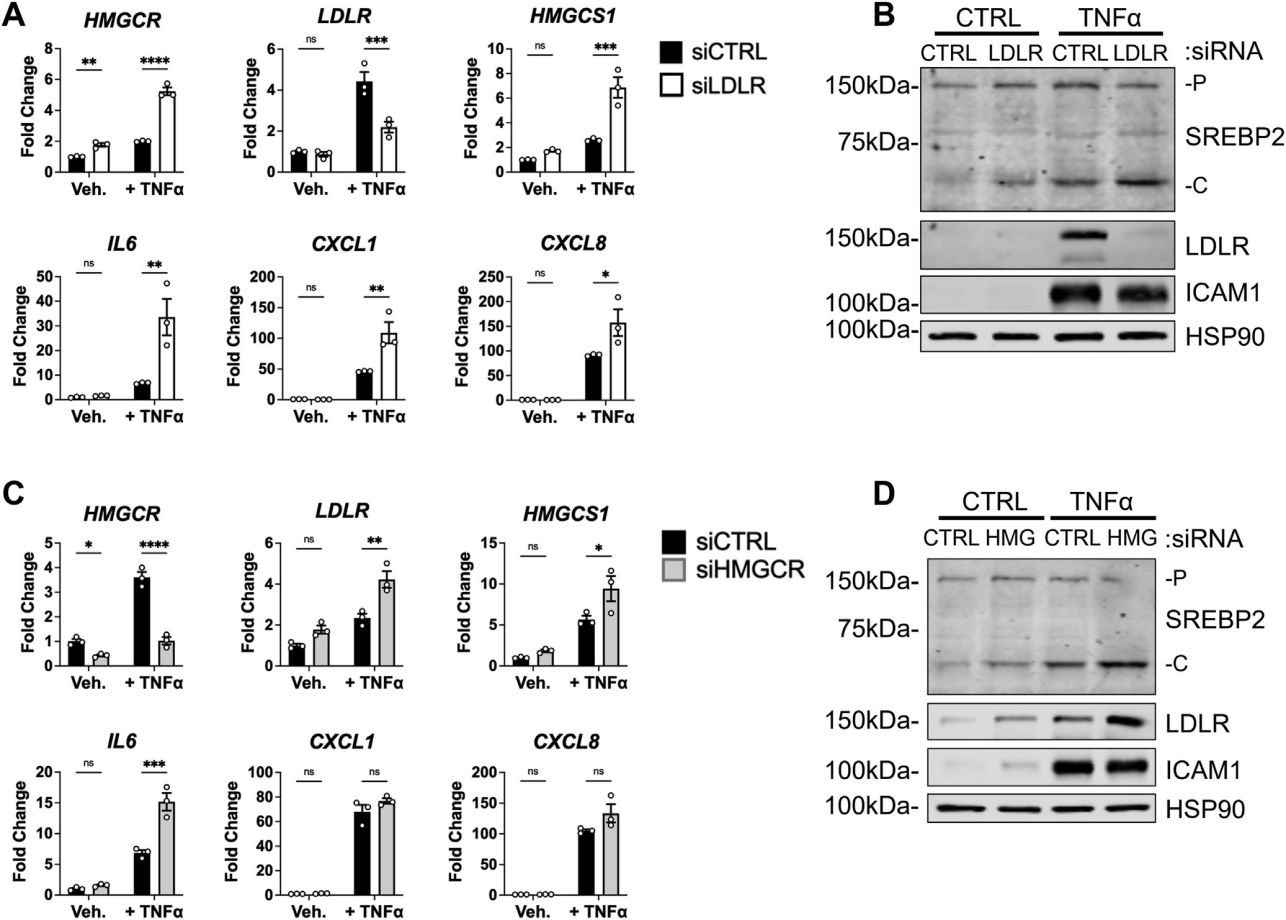
Knockdown of key proteins involved in exogenous cholesterol uptake or endogenous cholesterol synthesis increase chemokine expression in ECs under inflammatory stress. A: qRT-PCR analysis of RNA from HUVEC incubated in fetal bovine serum (FBS) for 24 h and treated with *LDLR* siRNA and with or without TNFα for an additional 16 h (10 ng/ml). Data are normalized to respective *ACTB* and then to untreated cells (n = 3). B: representative immunoblot of SREBP2-P (precursor), SREBP2-C (cleaved), and LDLR protein levels in HUVEC incubated in FBS for 24 h, treated with *LDLR* siRNA, and with or without TNFα for an additional 16 h (10 ng/ml). (n = 3). C: qRT-PCR analysis of RNA from HUVEC incubated in lipoprotein-depleted serum (LPDS) for 24 h and treated with *HMGCR* siRNA and with or without TNFα for an additional 16 h (10 ng/ml). Data are normalized to respective *ACTB* and then to untreated cells (n = 3). D: representative immunoblot of SREBP2-P (precursor), SREBP2-C (cleaved) , and LDLR protein levels in HUVEC incubated in LPDS for 16 h, treated with *HMGCR* siRNA, and with or without TNFα for an additional 16 h (10 ng/ml). (n = 3). ∗*P* < 0.05; ∗∗*P* < 0.01; ∗∗∗*P* < 0.001; ∗∗∗*P* < 0.0001 by one-way ANOVA with Tukey’s multiple comparison test.

**Fig. 5:**
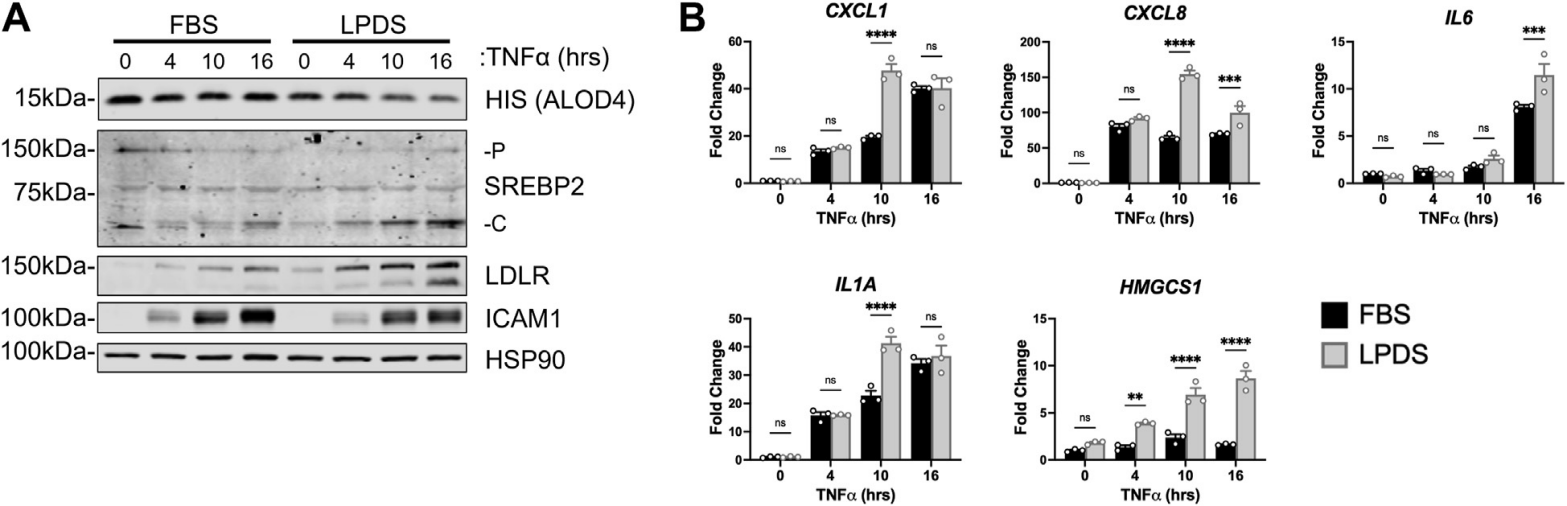
Restriction of exogenous lipoproteins upregulates pro-inflammatory chemokine transcription in cytokine-treated ECs. A: representative immunoblot of ALOD4, SREBP2-P (precursor), SREBP2-C (cleaved), and LDLR protein levels in HUVEC incubated in fetal bovine serum (FBS) or lipoprotein depleted serum (LPDS) for 24 h and treated with TNFα (10 ng/ml) for indicated time. B: qRT-PCR analysis of RNA from HUVEC incubated in FBS or LPDS for 24 h and treated with TNFα (10 ng/ml) for indicated time. Data are normalized to respective *ACTB* and then to untreated cells (n = 3). ∗*P* < 0.05; ∗∗*P* < 0.01; ∗∗∗*P* < 0.001; ∗∗∗*P* < 0.0001 by two-way ANOVA with Sidak’s multiple comparisons test.

We were particularly interested in the decreased expression of several chemokines that coincided with the loss of SREBP2. Therefore, we explored if SREBP2 regulated this gene set via modulation of cholesterol flux in ECs. Thus, we silenced the major regulator of exogenous cholesterol uptake, low-density lipoprotein receptor (*LDLR*), and the rate-limiting step of cholesterol biosynthesis, 3-hydroxy-3-methylglutaryl-coenzyme A reductase (*HMGCR*). RNA silencing of *LDLR* significantly decreased *LDLR* transcript and protein levels in the presence of full serum media (Fig. 4, A and B). When ECs were treated with TNFα, the loss of LDLR led to an increase in *IL6*, *CXCL1*, and *CXCL8* mRNA expression. *HMGCR* knockdown in sterol-depleted media also significantly upregulated SREBP2 activation and SREBP2-dependent gene transcription, as expected (Fig. 4, C and D). Interestingly, *HMGCR* knockdown also led to a significant increase in *IL6* expression and a trending increase in *CXCL8* expression. Knockdown of *LDLR* and *HMGCR* inhibit the replenishment of cellular cholesterol via lipoproteins and endogenous synthesis, however, they activate SREBP2. Similarly, cells treated with lipoprotein depleted serum (LPDS) decreased accessible cholesterol levels, increased SREBP2 activation, and significantly upregulated the expression of *CXCL1*, *CXCL8*, *IL6*, and *IL1A* when compared to cells in sterol-rich FBS (Fig. 5, A and B). Although our data indicate that SREBP2 feeds forward into the pro-inflammatory phenotype of ECs, the limitation of both de novo cholesterol synthesis and exogenous uptake produced the same directionality in EC inflammatory phenotype. Both perturbations increase SREBP2 activity via the canonical negative feedback loop of post-translational SREBP2 processing. Therefore, SREBP2 DNA-binding and transcriptional activity may be the prominent regulatory mechanism for enhanced inflammatory chemokine expression in EC.

### SREBP2 chromatin immunoprecipitation sequencing reveals significant binding to *BHLHE40* and *KLF6* gene loci, two non-classical SREBP2 targets

**Fig. 6:**
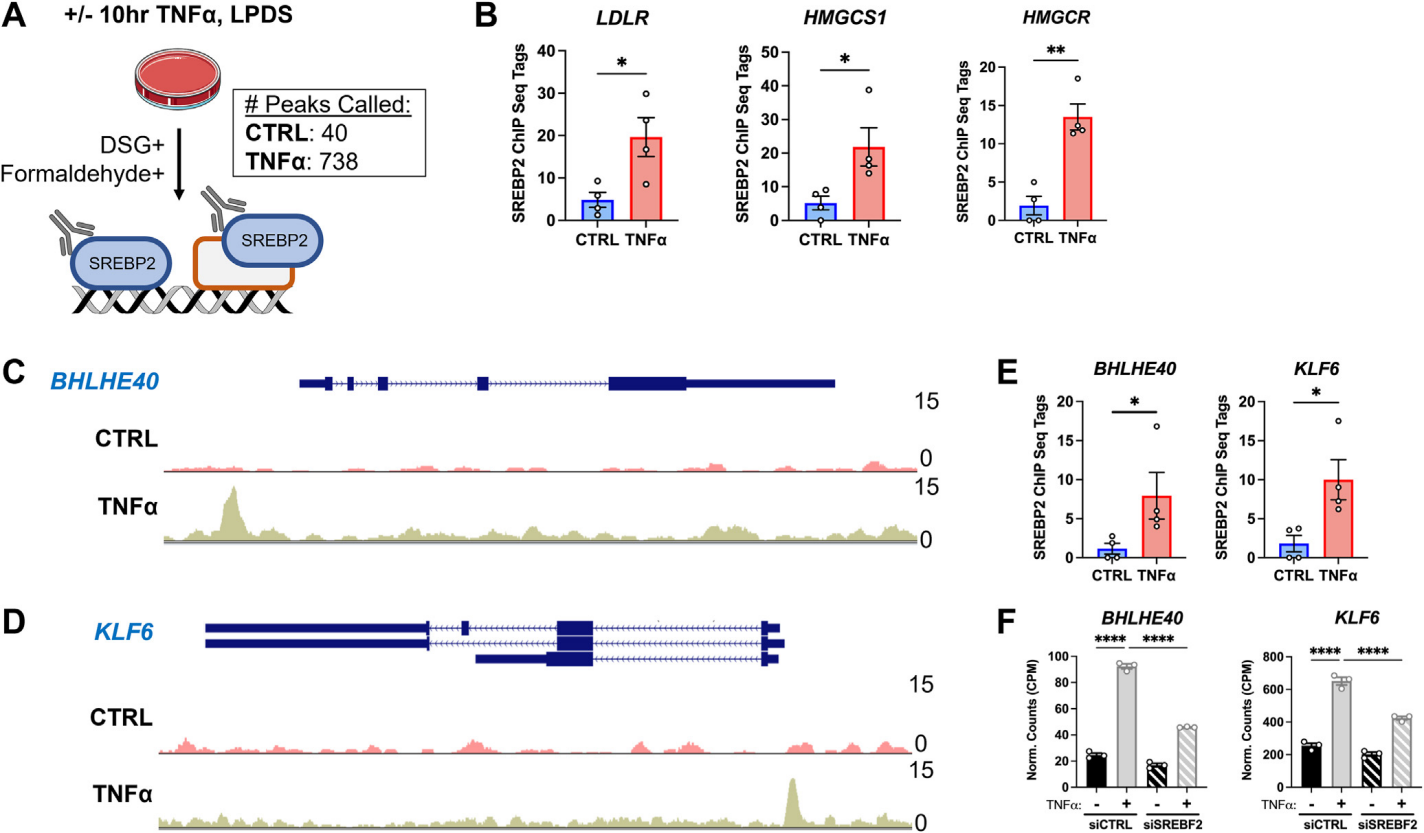
Endogenous SREBP2 ChIP-seq in ECs treated with TNFα. A: schematic of SREBP2 ChIP-seq protocol in HUVEC treated with or without TNFα (10 ng/ml) for 10 h in lipoprotein-depleted serum (LPDS) (n = 4). B: quantification of representative ChIP-seq tags for *LDLR*, *HMGCS1*, and *HMGCR* gene loci from (A). C: SREBP2 binding to *BHLHE40* gene locus. D: SREBP2 binding to *KLF6* gene locus. E: quantification of ChIP-seq tags for *BHLHE40* and *KLF6* gene loci from (A). F: *BHLHE40* and *KLF6* expression from previous RNA-seq experiments (15). Data for ChIP-seq analysis is available in GEO under accession code GSE223094 and RNA-seq analysis is available in GEO under accession code GSE201466. Data for (D) and (E) are scaled from 0 (bottom) to 15 (top). ∗*P* < 0.05; ∗∗*P* < 0.01; ∗∗∗*P* < 0.001; ∗∗∗*P* < 0.0001 by one-way ANOVA with Tukey’s multiple comparison test (F) or *t* test (B and E).

The RNA sequencing data indicated that activation of SREBP2 may contribute to the transcriptional regulation of several pro-inflammatory chemokines in TNFα-treated ECs. SREBP2 is a transcription factor that classically upregulates cholesterol biosynthesis genes, but recent reports have also found that it can transcriptionally activate pro-inflammatory genes, such as *NLRP3*, *NOX2*, *PTGS2*, *IL8*, and several other pro-inflammatory pathways (5, 11, 21, 22). Therefore, we performed SREBP2 chromatin immunoprecipitation sequencing (ChIP-seq) on ECs treated with or without TNFα for 10 h. We fixed cells with formaldehyde and disuccinimidyl glutarate (DSG) to capture DNA bound to SREBP2 or bound to another protein in a complex with SREBP2. As expected, TNFα treatment resulted in a greater number of called peaks (748 peaks) compared to control (40 peaks) (Fig. 6A). Among the top 50 hits, an overwhelming number of genes belonged to classical SREBP2-dependent pathways, such as *LDLR*, *HMGCS1*, and *HMGCR* (supplemental data S1A). Furthermore, SREBP2 binding to all sterol-sensitive gene loci was significantly upregulated by TNFα (Fig. 6B). Analysis of the *LDLR* promoter revealed a characteristic dual binding peak near the promoter region, which has been reported in previous SREBP2 ChIP-seq experiments in the ENCODE Database (supplemental data S1B).

SREBP2 ChIP-seq dataset was probed for overrepresented motifs using Homer de novo motif analysis. SREBF1 was predicted for the gene set within 3.93% of targets (supplemental data S1C). Furthermore, NFY is a transcription factor reported to facilitate SREBP activity in the nucleus and acts as a coactivator of sterol-sensitive genes (23). NFY motifs were found in 20.46% of peaks. Lastly, several other transcription factor motifs were found from SREBP2 ChIP-seq peaks, such as the pro-inflammatory factors AP1 and REL, which were found in 10.03% and 4.20% of targets, respectively. This suggests that SREBP2 may perform nonclassical transcriptional activation in the context of inflammatory stress.

Two gene targets of interest were identified from the SREBP2 ChIP-seq dataset as possibly playing a role in the EC inflammatory phenotype: *BHLHE40* and *KLF6*. Both genes encode transcription factors that have been previously reported to promote pro-inflammatory phenotypes in T-cells and macrophages (24, 25). *BHLHE40* and *KLF6* gene loci had high signal-to-noise for SREBP2 pull-down and were both within the top 50 peaks called (supplemental data S1A). Detailed analysis of the promoter region of *BHLHE40 and KLF6* revealed prominent SREBP2 binding peaks within their respective promoter regions. (Fig. 6, C and D). Second, both genes contained higher ChIP-seq tags in TNFα-treated cells compared to the control (Fig. 6E). Importantly, these two genes were significantly upregulated by TNFα and inhibited by the loss of *SREBF2* from RNA-seq results in HUVEC (Fig. 6F). This supported the idea that their role in the EC inflammatory response was at least partially through SREBP2 activation.

### Expression of constitutively active N-terminal SREBP2 is sufficient to significantly upregulate the expression of *BHLHE40* and *KLF6*

**Fig. 7:**
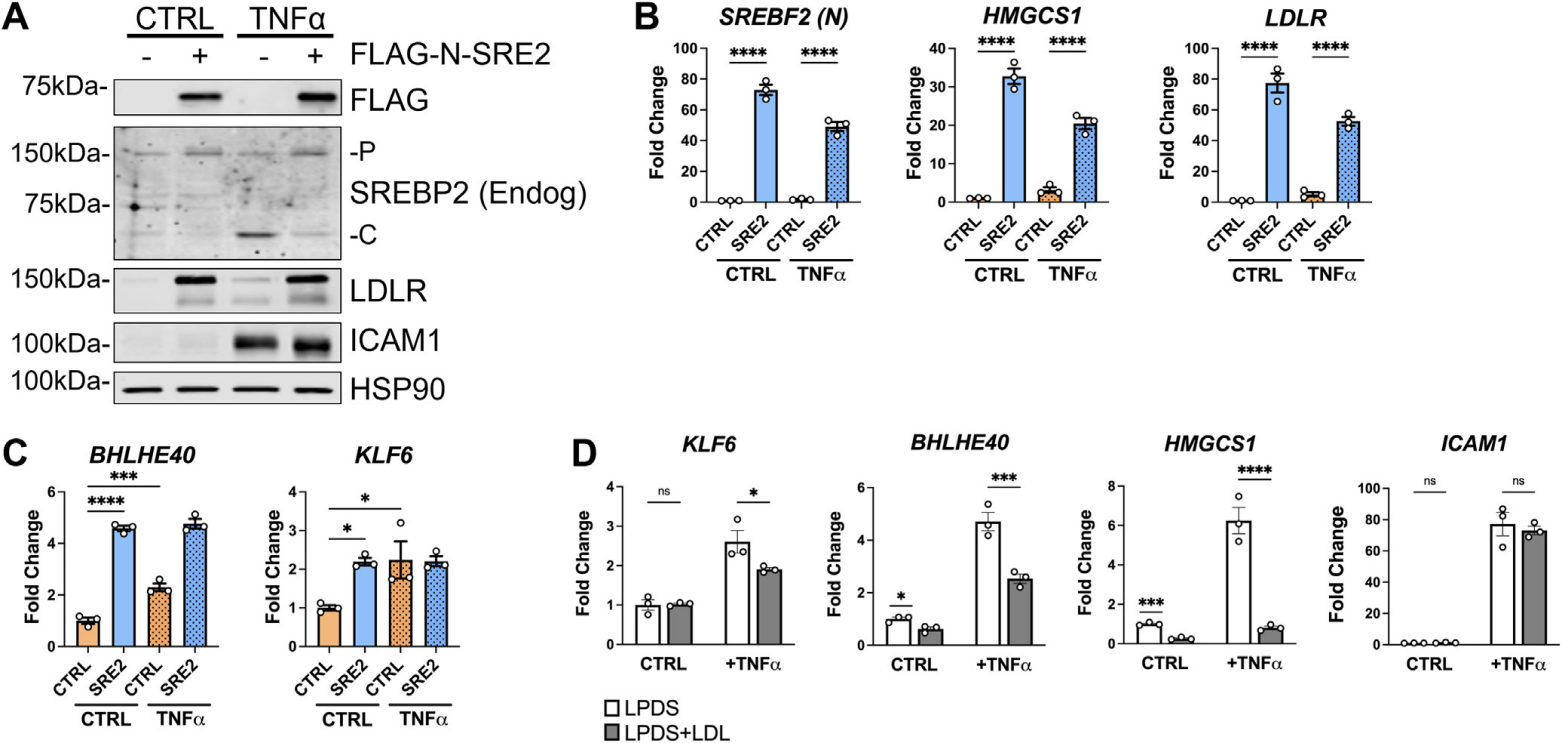
Lentiviral expression of constitutively active N-terminal SREBP2 is sufficient to upregulate *BHLHE40* and *KLF6* levels. A: representative immunoblot of FLAG (N-SREBP2), endogenous SREBP2 (endog), and LDLR protein levels in HUVEC expressing lentiviral-driven FLAG-N-SREBP2 (FLAG-N-SRE2) and treated with or without TNFα (10 ng/ml). Negative controls were treated with empty vector lentivirus. B: qRT-PCR analysis of classical SREBP2-dependent gene expression in HUVEC expressing lentiviral-driven FLAG-N-SREBP2 (S2) and treated with or without TNFα (10 ng/ml). Data are normalized to respective *ACTB* and then to untreated cells (n = 3). C: qRT-PCR analysis of *BHLHE40* and *KLF6* expression in HUVEC expressing lentiviral-driven FLAG-N-SREBP2 (S2) and treated with or without TNFα (10 ng/ml). Data are normalized to respective *ACTB* and then to untreated cells (n = 3). D: qRT-PCR analysis of *KLF6*, BHLHE40, *HMGCS1*, and *ICAM1* expression in HUVEC treated with or without TNFα (10 ng/ml) after overnight incubation in lipoprotein-depleted serum (LPDS) or LPDS+LDL (50 μg/ml). (n = 3). ∗*P* < 0.05; ∗∗*P* < 0.01; ∗∗∗*P* < 0.001; ∗∗∗*P* < 0.0001 by one-way ANOVA with Tukey’s multiple comparison test (B) and (C) or two-way ANOVA with Sidak’s multiple comparisons test (D).

Because little is known about the role of BHLHE40 and KLF6 in EC biology and these targets have not been extensively studied as sterol-sensitive factors, we expressed a constitutively active form of SREBP2 into HUVEC to independently verify the ChIP-seq data. The first 470 amino acids of *SREBF2* were cloned into the lentiviral pSMPP expression factor with a DYKDDDDK (FLAG) tag attached to the 5′ end of the gene (FLAG-N-SREBP2). When expressed into HUVEC, this construct produced a FLAG-tagged DNA binding domain of SREBP2 that cannot be inhibited by cholesterol (Fig. 7A). As a positive control, FLAG-N-SREBP2 (FLAG-N-SRE2) increased LDLR protein levels, as well as transcription of *HMGCS1* and *LDLR* (Fig. 7, A and B) whereas the empty vector did not. FLAG-N-SRE2 also inhibited TNFα-induced endogenous SREBP2 activation due to upregulation of cholesterol biosynthesis and uptake. Furthermore, FLAG-N-SRE2 significantly amplified transcription of *BHLHE40* and *KLF6* greater than or equal to levels after TNFα treatment (Fig. 7C). These results indicated that *BHLHE40* and *KLF6* expression should be sensitive to sterol depletion and saturation. Indeed, *BHLHE40* and *KLF6* transcripts were significantly inhibited in HUVEC after supplementation with high levels of low-density lipoprotein (LDL), which were sufficient to decrease classical SREBP2 target genes, such as *HMGCS1* (Fig. 7D). Therefore, *BHLHE40* and *KLF6* may be novel genes regulated by SREBP2 and may play a role in EC inflammatory gene expression.

### *KLF6* knockdown significantly attenuates the expression of pro-inflammatory chemokines similar to *SREBF2* knockdown

**Fig. 8:**
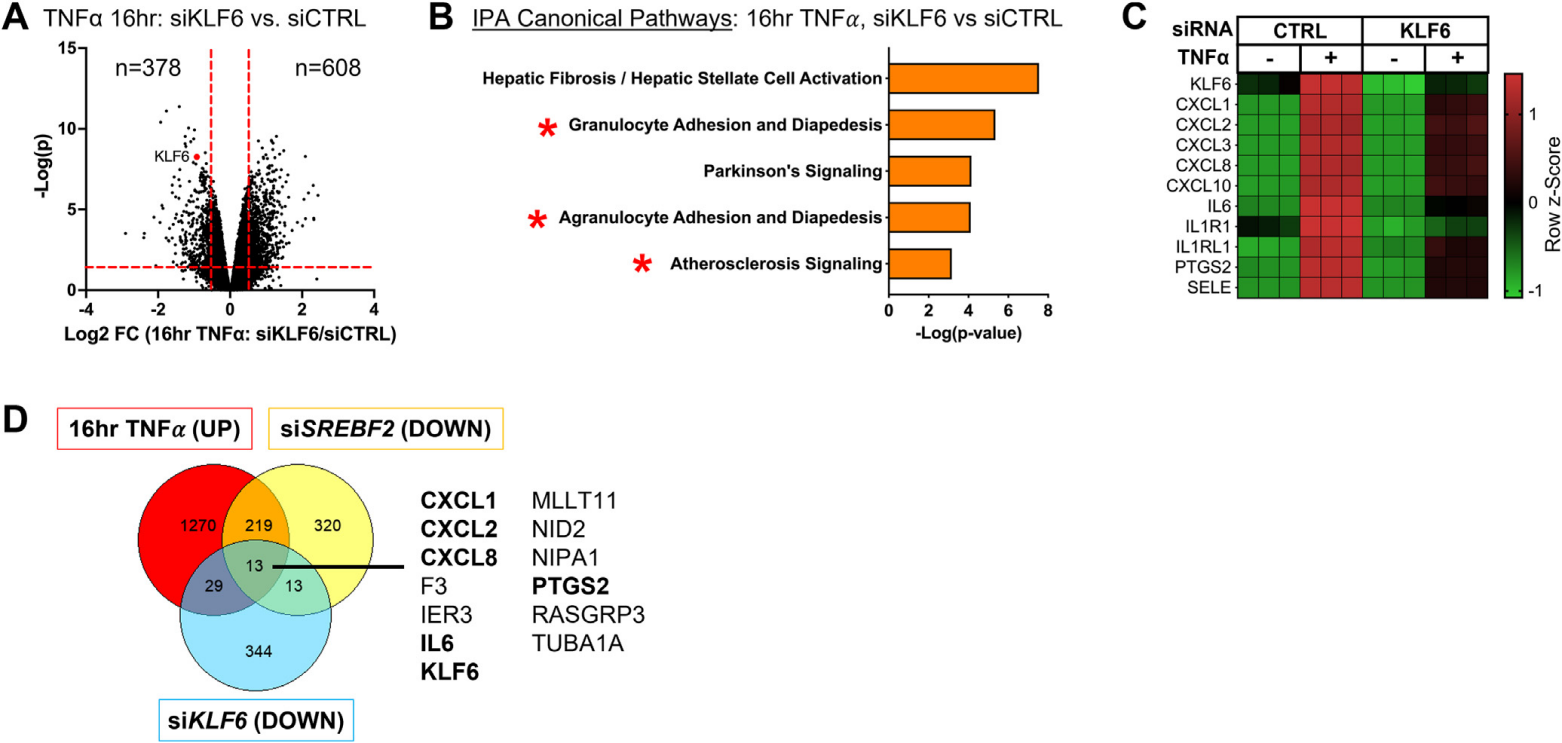
RNAseq analysis of KLF6 knockdown in ECs reveals a significant inhibition chemokine gene expression that shares overlap with *SREBF2* knockdown. A: volcano plot of RNA-seq analysis of differentially expressed genes of HUVEC treated with TNFα (10 ng/ml) for 16 h and with or without siRNA targeting *CTRL* or *KLF6*. Red lines indicate cutoffs used for pathway analysis (−1.5<F.C.<1.5; *P* < 0.05). B: ingenuity pathway analysis for pathways significantly decreased with KLF6 knockdown using genes from (A). C: heatmap of representative genes from (A) showing three independent donors. D: Venn diagram of gene overlap between 3 sets of RNA seq experiments, genes significantly increased by TNFα treatment, decreased by *SREBF2* knockdown, and decreased by *KLF6* knockdown. Data represent the analysis of three independent donor lines. Data are available in GEO under accession code GSE207919. ∗*P* < 0.05; ∗∗*P* < 0.01; ∗∗∗*P* < 0.001; ∗∗∗*P* < 0.0001 by one-way ANOVA with Tukey’s multiple comparison test.

Several pieces of data led to the prioritization of KLF6 as a top candidate that could bridge the mechanism of SREBP2 activation and inflammatory chemokine expression. First, early work in epithelial cells and macrophages clearly indicated that KLF6 transactivates NF-κB to specifically regulate the expression of a select subset of pro-inflammatory genes, mainly chemokines (26). Second, RNA sequencing data from our laboratory revealed that *KLF6* was significantly upregulated at 4 and 10 h TNFα treatment (GSE201466). Furthermore, RNA sequencing data indicated that *KLF6* was the highest expressed member of the KLF family in HUVEC at baseline. Therefore, we performed RNA sequencing analysis on HUVEC treated with TNFα for 16 h and siRNA targeting *KLF6*. In ECs treated with TNFα, *KLF6* knockdown significantly inhibited 378 genes and activated 608 genes (*P* < 0.05, −1.5 > F.C > 1.5) (Fig. 8A). Canonical Pathway analysis on genes significantly downregulated with the loss of KLF6 revealed significant inhibition of “Granulocyte Adhesion and Diapedesis” as well as “Atherosclerosis Signaling” pathways (Fig. 8B). Indeed, these pathways contained genes that were shared by sequencing data of *SREBF2* knockdown, such as *CXCL1*, *CXCL8*, *IL6*, and prostaglandin G/H synthase 2 (*PTGS2*) (Fig. 8C). Comparison of the data from the two RNA sequencing experiments of targets that were significantly upregulated by TNFα, significantly downregulated by si*SREBF2*, and significantly downregulated by *siKLF6*, reveal a distinct set of 13 genes that included several chemokine family members (Fig. 8D).

## Discussion

Our previous study identified SREBP2 as an active pathway in the late phase of EC acute inflammatory response and here we report that SREBP2 contributes to the overall EC inflammatory response. We found that loss of SREBP2 produced a specific transcriptomic response in ECs treated with TNFα, including decreased chemokine expression and upregulation of the type I interferon response. Our evidence suggested that SREBP2’s effect on chemokine expression was not regulated by cholesterol flux, but, rather, through direct gene transcription. We performed the first endogenous SREBP2 ChIP-seq in ECs and found *BHLHE40* and *KLF6* as two novel targets of SREBP2 binding. Of these two targets, we found that KLF6 influences the expression of the same chemokine gene sets as identified in SREBF2 knockdown RNA sequencing. These results solidify the SREBP2 pathway to be actively involved in cellular inflammatory responses.

Interestingly, we found that the flux of cholesterol did not contribute to the regulation of chemokine expression by SREBP2. Numerous studies have reported that aberrant activation of cholesterol biosynthesis or uptake may lead to upregulation of inflammatory signaling, activation of the inflammasome, or positive feedback towards trained immunity (7, 8, 9). However, in our hands, limiting cholesterol flux by restricting exogenous lipoproteins or knockdown of key proteins involved in cholesterol biosynthesis and uptake significantly increased the expression of chemokines. Indeed, the effect of inflammatory stress on total cholesterol overload may not be appreciable in the shorter time frame of this study and may need to be explored in the context of days, not hours. EC cholesterol homeostasis in atherosclerosis presents an interesting mechanistic quandary because ECs in this disease pathology are both chronically inflamed, yet directly exposed to elevated levels of cholesterol in the bloodstream. Therefore, progressive cholesterol overload may play a larger role in EC phenotype.

This study reports endogenous SREBP2 ChIP-seq in ECs and found that ECs treated with TNFα caused SREBP2 to bind to a greater coverage of gene loci than untreated cells. Other studies have utilized SREBP2 ChIP to show that it was bound to the promoter of pro-inflammatory genes. First, it was found that SREBP2 could bind to the promoter of *NLRP3* and *NOX2*, which was responsible for shear stress-induced activation of the inflammasome in ECs (11). More recently, Kusnadi *et al.* (5) showed SREBP2 bound to the promoters of several pro-inflammatory genes, such as *IL1B* and *CXCL10*, in macrophages treated with TNFα. However, we did not observe direct SREBP2 binding to any of these pro-inflammatory gene loci. Notably, these studies were done in cells overexpressing constitutively active N-SREBP2, not via endogenous levels of SREBP2. Although overexpression allows for more effective SREBP2 pull-down, it also comes at the cost of SREBP2 expression in the nucleus far beyond the physiological levels, which has the potential to produce spurious SREBP2 binding events and false positives. Here, we report the first endogenous SREBP2 ChIP-seq performed in human cells under inflammatory stress. Although the coverage was notably smaller than in previous publications, many of the top genes identified belonged to the cholesterol biosynthetic pathway, serving as a robust positive control and strengthening the confidence in the results.

TNFα treatment caused SREBP2 to significantly bind to the promoters of two pro-inflammatory transcription factors, *BHLHE40* and *KLF6*. Furthermore, this finding was supported by our previous RNA-seq data that indicated a loss of SREBP2 significantly decreased the expression of these two genes. Lastly, expression of constitutively active N-SREBP2 was sufficient to upregulate *BHLHE40* and *KLF6*, confirming that these two genes could be directly transcriptionally activated by SREBP2. Although little is known about these two transcription factors in ECs, previous reports have characterized their pro-inflammatory roles in leukocytes. Firstly, BHLHE40 was shown to direct T cells towards a more pro-inflammatory phenotype and away from immune tolerance by directly regulating inflammatory genes, such as granulocyte-macrophage colony-stimulating factor 2 (*CSF2*), *PTGS2*, interleukin-1 alpha (*IL1A*), and interleukin-17A (*IL17A*) (27). Interestingly, BHLHE40 has been reported as a transcription factor required for hepatic SREBP1c upregulation in response to insulin, indicating that there may be a feedback loop between lipid homeostasis, SREBP, and BHLHE40 (28). Expression of KLF6 was shown to promote macrophage polarization and co-activate specific NF-κB genes *CXCL2*, *CXCL8*, *IL1A*, and interleukin-1 beta (*IL1B*) (26, 29). Several of these same genes were also significantly downregulated by *SREBF2* knockdown in our data and, indeed, *KLF6* knockdown significantly inhibited the expression of these chemokines in ECs. Endothelial KLF6 warrants an even deeper investigation and may be an important part of the mechanism that links cholesterol homeostasis to the inflammatory response.

This study builds on a growing body of work indicating a pro-inflammatory role of SREBP2 in inflammation and immunity. Here, we focused on the endothelium and find that SREBP2 upregulates novel transcription factors involved in the inflammatory process providing strong justification for further exploration of the role of EC SREBP2 in diseases of chronic inflammation, such as atherosclerosis, a pathology that bridges imbalances in cholesterol and inflammation.

## Data availability

The RNA-seq data sets were deposited in Gene Expression Omnibus (GEO, NCBI) public repository, GEO accession numbers GSE207787 (siSREBP2) and GSE207919 (siKLF6). ChIP-seq data sets were also deposited under GEO accession number GSE223094.

## Supplemental data

This article contains supplemental data.

## Conflict of interest

The authors declare no competing interests.

## Figures and Tables

**Supplemental Data S1:**
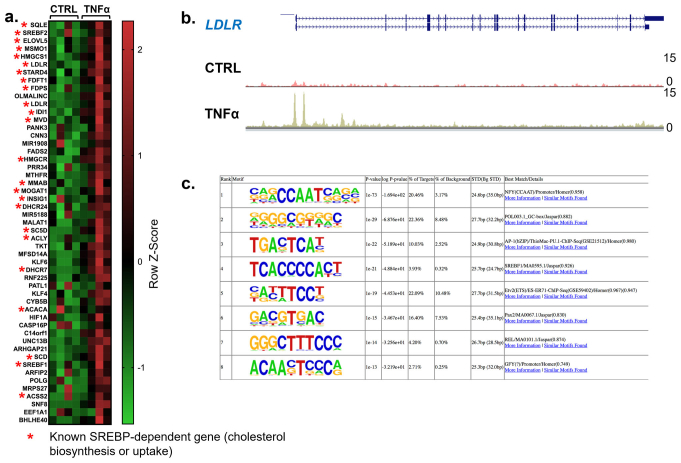
Expanded data and motif analysis of SREBP2 ChIP-seq. A: Heatmap of 50 top genes from Fig. 4A. Red asterisk indicates classical SREBP2 genes previously reported in literature. B: SREBP2 binding to LDLR gene locus from Fig. 4A. Data are scaled from 0 (bottom) to 15 (top). C: The top eight enriched motifs that occur in enhancers from previous SREBP2 ChIP-seq in HUVEC treated with TNFa (10 ng/mL). The transcription factor (TF) family, de novo motif matrix, percentage motif occurrence at enhancer loci versus random loci, and enrichment –log(p) values are indicated. Enrichment was calculated from 200 bp sequence, centered on chromatin accessibility.

## Supplemental data

Supplemental Data S2 RNA sequencing and ChIP sequencing output data. Normalized counts for siSREBF2 and siKLF6. SREBP2 ChIP-seq information.

Supplemental Data S3 Table of reagents.
