# Supplementary material for: SREBP2 regulates the endothelial response to cytokines via direct transcriptional activation of KLF6
Source: J Lipid Res. 2023 Jul 10;64(8):100411. doi: 10.1016/j.jlr.2023.100411 (PMC10407908; doi:10.1016/j.jlr.2023.100411)
Supplement: Supplemental Data S3 — Table of reagents. [file mmc2.docx]

| **Key Resources Table** | | | | |
| --- | --- | --- | --- | --- |
| **Reagent type (species) or resource** | **Designation** | **Source or reference** | **Identifiers** | **Additional information** |
| antibody | Anti-His Tag Dylight 680 (Mouse monoclonal) | abcam | ab18184 | WB (1:1,000) |
| antibody | DYKDDDDK Antibody (Rabbit monoclonal) | Cell Signaling | 2368S | WB (1:1,000) |
| antibody | FITC-hsICAM1 (Mouse monoclonal) | Biolegend | 322720 | FACS (1:100) |
| antibody | Anti-HSP90 (Mouse monoclonal) | Santa Cruz | sc-13119 | WB (1:2,000) |
| antibody | Anti-ICAM1 (Rabbit monoclonal) | Cell Signaling | 4915S | WB (1:1,000) |
| antibody | Anti-LDLR (Rabbit monoclonal) | abcam | ab52818 | WB (1:1,000) |
| antibody | Anti-P65/RELA (Rabbit monoclonal) | Cell Signaling | 8242S | WB (1:1,000) |
| antibody | Pacific Blue-hsHLA-A,B,C (Mouse monoclonal) | Biolegend | 311418 | FACS (1:100) |
| antibody | PE-hsVCAM1 (Mouse monoclonal) | Biolegend | 305806 | FACS (1:100) |
| antibody | Anti-SREBP2 (Mouse monoclonal) | BD Biosciences | 557037 | WB (1:1,000) |
| antibody | Anti-VCAM1 (Mouse monoclonal) | Santa Cruz | sc-13160 | WB (1:1,000) |
| sequence-based reagent | siRNA: *RELA* | Thermofisher Scientific | s11914 | Silencer Select |
| sequence-based reagent | siRNA: *SREBF2* | Thermofisher Scientific | s27 | Silencer Select |
| sequence-based reagent | siRNA: *HMGCR* | Thermofisher Scientific | 110740 | Silencer |
| sequence-based reagent | siRNA: *SCAP* | Thermofisher Scientific | s695 | Silencer Select |
| sequence-based reagent | siRNA: *KLF6* #1 | Dharmacon | D-021331-01-0002 |  |
| sequence-based reagent | siRNA: *KLF6* #2 | Dharmacon | D-021331-03-0002 |  |
| sequence-based reagent | hs*ACTB*_F | Fowler *et al.*, 2022 | qRT-PCR Primers | AGCACTGTGTTGGCGTACAG |
| sequence-based reagent | hs*ACTB*_R | Fowler *et al.*, 2022 | qRT-PCR Primers | GGACTTCGAGCAAGAGATGG |
| sequence-based reagent | hs*LDLR*_F | Fowler *et al.*, 2022 | qRT-PCR Primers | TCTGCAACATGGCTAGAGACT |
| sequence-based reagent | hs*LDLR*_R | Fowler *et al.*, 2022 | qRT-PCR Primers | TCCAAGCATTCGTTGGTCCC |
| sequence-based reagent | hs*HMGCS1*_F | Fowler *et al.*, 2022 | qRT-PCR Primers | CAAAAAGATCCATGCCCAGT |
| sequence-based reagent | hs*HMGCS1*_R | Fowler *et al.*, 2022 | qRT-PCR Primers | AAAGGCTTCCAGGCCACTAT |
| sequence-based reagent | hs*HMGCR*_F | Fowler *et al.*, 2022 | qRT-PCR Primers | TGATTGACCTTTCCAGAGCAAG |
| sequence-based reagent | hsI*NSIG1*_F | Fowler *et al.*, 2022 | qRT-PCR Primers | CTAAAATTGCCATTCCACGAGC |
| sequence-based reagent | hs*INSIG1*_R | Fowler *et al.*, 2022 | qRT-PCR Primers | GCACTGCATTAAACGTGTGG |
| sequence-based reagent | hs*SREBF2*_F | Fowler *et al.*, 2022 | qRT-PCR Primers | TAAAGGAGAGGCACAGGA |
| sequence-based reagent | hs*SREBF2*_R | Fowler *et al.*, 2022 | qRT-PCR Primers | AGGAGAACATGGTGCTGA |
| sequence-based reagent | hs*ICAM1*_F | Fowler *et al.*, 2022 | qRT-PCR Primers | GTGGTAGCAGCCGCAGTC |
| sequence-based reagent | hs*ICAM1*_R | Fowler *et al.*, 2022 | qRT-PCR Primers | GGCTTGTGTGTTCGGTTTCA |
| sequence-based reagent | hs*CXCL1*_F | Fowler *et al.*, 2022 | qRT-PCR Primers | AGGGAATTCACCCCAAGAAC |
| sequence-based reagent | hs*CXCL1*_R | Fowler *et al.*, 2022 | qRT-PCR Primers | TGGATTTGTCACTGTTCAGCA |
| sequence-based reagent | hs*SELE*_F | Fowler *et al.*, 2022 | qRT-PCR Primers | ACCTCCACGGAAGCTATGACT |
| sequence-based reagent | hs*SELE*_R | Fowler *et al.*, 2022 | qRT-PCR Primers | CAGACCCACACATTGTTGACTT |
| sequence-based reagent | hs*SCAP*_F | Fowler *et al.*, 2022 | qRT-PCR Primers | CGCAAACAAGGAGAGCCTAC |
| sequence-based reagent | hs*SCAP*_R | Fowler *et al.*, 2022 | qRT-PCR Primers | TGTCTCTCAGCACGTGGTTC |
| sequence-based reagent | hsCXCL8_F | This Paper | qRT-PCR Primers | GTGCAGTTTTGCCAAGGAGT |
| sequence-based reagent | hsCXCL8_R | This Paper | qRT-PCR Primers | CTCTGCACCCAGTTTTCCTT |
| sequence-based reagent | hsIL6_F | This Paper | qRT-PCR Primers | TACCCCCAGGAGAAGATTCC |
| sequence-based reagent | hsIL6_R | This Paper | qRT-PCR Primers | TTTTCTGCCAGTGCCTCTTT |
| sequence-based reagent | hsPTGS2_F | This Paper | qRT-PCR Primers | TGAAACCCACTCCAAACACA |
| sequence-based reagent | hsPTGS2_R | This Paper | qRT-PCR Primers | GAGAAGGCTTCCCAGCTTTT |
| sequence-based reagent | hsIL1A_F | This Paper | qRT-PCR Primers | AATGACGCCCTCAATCAAAG |
| sequence-based reagent | hsIL1A_R | This Paper | qRT-PCR Primers | TGGGTATCTCAGGCATCTCC |
| sequence-based reagent | hsBHLHE40_F | This Paper | qRT-PCR Primers | CCTTGAAGCATGTGAAAGCA |
| sequence-based reagent | hsBHLHE40_R | This Paper | qRT-PCR Primers | GCTTGGCCAGATACTGAAGC |
| sequence-based reagent | hsKLF6_F | This Paper | qRT-PCR Primers | CACGAGACCGGCTACTTCTC |
| sequence-based reagent | hsKLF6_R | This Paper | qRT-PCR Primers | CGGATTCCTCCTTTTTCTCC |
| sequence-based reagent | FLAG-N-SREBF2 Lenti Cloning_F | This Paper | Cloning Primer | cgacagactgagtcgcccgggggggatccCAAGatggattacaaggatgacgacgataaggattacaaggatgacgacgataag |
| sequence-based reagent | FLAG-N-SREBF2 Lenti Cloning_R | This Paper | Cloning Primer | gcctgcaggtcgactctagagtcgcggccgcttaaacgggccctctagagtcgag |
| sequence-based reagent | pSMPP lentiviral backbone | Addgene | 104970 |  |
| chemical compound, drug | 25-hydroxycholesterol | Sigma Aldrich | H1015 |  |
| chemical compound, drug | EGM2 | Lonza | CC-3162 |  |
| chemical compound, drug | Lipoprotein Depleted Serum (LPDS) | Kalen Biomedical | 880100 |  |
| chemical compound, drug | Native LDL | Kalen Biomedical | 770200 |  |
| chemical compound, drug | rhTNFα | RD Systems | 210-TA-020/CF |  |
| software, algorithm | Partek Flow | Partek | https://www.partek.com/partek-flow/ |  |
| software, algorithm | Ingenuity Pathway Analssis | Qiagen | https://digitalinsights.qiagen.com/products-overview/discovery-insights-portfolio/analysis-and-visualization/qiagen-ipa/ |  |
